# Burnout syndrome in Brazilian and Spanish nursing workers*

**DOI:** 10.1590/1518-8345.2818.3192

**Published:** 2019-12-05

**Authors:** Maria Baldonedo-Mosteiro, Mirian Cristina dos Santos Almeida, Patricia Campos Pavan Baptista, Marta Sánchez-Zaballos, Francisco Javier Rodriguez-Diaz, Maria Pilar Mosteiro-Diaz

**Affiliations:** 1 Universidade de Oviedo, Departamento de Psicologia, Oviedo, Astúrias, Spain.; 2 Universidade Federal do Tocantins (UFT), Curso de Enfermagem, Palmas, TO, Brazil.; 3 Scholarship holder at the Coordenação de Aperfeiçoamento de Pessoal de Nível Superior (CAPES), Brazil.; 4 Universidade de São Paulo (USP), Escola de Enfermagem, São Paulo, SP, Brazil.; 5 Universidade de Oviedo, Departamento de Medicina, Oviedo, Astúrias, Spain.

**Keywords:** Burnout, Professional, Despersonalization, Nursing, Nursing Staff, Health Personnel Management, Occupational Health, Esgotamento Profissional, Despersonalização, Enfermagem, Recursos Humanos de Enfermagem, Administração de Recursos Humanos em Saúde, Saúde do Trabalhador, Agotamiento Profesional, Despersonalización, Enfermería, Personal de Enfermería, Administración del Personal en Salud, Salud Laboral

## Abstract

**Objective:**

to analyze the burnout dimensions scores in Brazilian and Spanish nursing workers.

**Method:**

quantitative, cross-sectional and comparative study conducted with 589 Nursing workers who answered the Sociodemographic and Professional Characterization Questionnaire and the Maslach Burnout Inventory. Descriptive and analytical analysis of the data was performed.

**Results:**

Spanish Nursing workers presented higher averages in the Depersonalization dimension (p = 0.004) and Brazilians, higher scores in the Professional Achievement dimension (p = 0.031). In both Spain and Brazil, nursing assistants / technicians were found to have higher Emotional Exhaustion than nurses; In Brazil, Depersonalization is higher in nurses and in Spain it is higher in Nursing assistants / technicians. Statistically significant results were found in the association of burnout dimensions with sociodemographic and work characteristics: age; professional category; workplace; work regime; work shift; time of professional experience; working time in the same workplace and consider stressful work.

**Conclusion:**

Although Brazilian and Spanish nursing workers score low levels of Depersonalization and high Professional Achievement, there are average levels of Emotional Exhaustion, indicating an important preventive factor to be worked on, since Emotional Exhaustion is considered the first stage of burnout.

## Abstract (pt)

**Objetivo:**

analisar os escores das dimensões do *burnout* em trabalhadores de enfermagem brasileiros e espanhóis.

**Método:**

estudo quantitativo, transversal e comparativo, realizado com 589 trabalhadores de enfermagem que responderam o Questionário de Caracterização Sociodemográfica e Profissional e o *Maslach Burnout Inventory*. Procedeu-se a análise descritiva e analítica dos dados.

**Resultados:**

Os trabalhadores de enfermagem espanhóis apresentam maiores médias na dimensão Despersonalização (p=0,004) e os brasileiros maiores pontuações na dimensão Realização Profissional (p=0,031). Observou-se que tanto na Espanha quanto no Brasil auxiliares/técnicos de enfermagem possuem maior Exaustão Emocional do que os enfermeiros; no Brasil a Despersonalização é maior em enfermeiros, na Espanha é maior em auxiliares/técnicos de enfermagem. Verificou-se resultados estatisticamente significativos na associação das dimensões do *burnout* com as características sociodemográficas e de trabalho: idade, categoria profissional, local de trabalho, regime de trabalho, turno de trabalho, tempo de experiência profissional, tempo de atuação no mesmo local de trabalho e considerar o trabalho estressante.

**Conclusão:**

apesar dos trabalhadores de enfermagem brasileiros e espanhóis pontuarem níveis baixos de Despersonalização e elevados de Realização Profissional, verifica-se níveis médios de Exaustão Emocional, indicando um fator preventivo importante a ser trabalhado, uma vez que a Exaustão Emocional é considerada o primeiro estágio do *burnout*.

## Abstract (es)

**Objetivo:**

analizar los puntajes de las dimensiones del burnout en trabajadores de enfermería brasileños y españoles.

**Método:**

estudio cuantitativo, transversal y comparativo realizado con 589 trabajadores de enfermería que respondieron el cuestionario de caracterización sociodemográfica y profesional y el *Maslach Burnout Inventory*. Se realizó un análisis descriptivo y analítico de los datos.

**Resultados:**

los trabajadores de enfermería españoles tuvieron promedios más altos en la dimensión de Despersonalización (p=0,004) y los brasileños más altos puntajes en la dimensión de Logro Profesional (p=0,031). Tanto en España como en Brasil, se descubrió que los asistentes/técnicos de enfermería tenían un mayor Agotamiento Emocional que los enfermeros; en Brasil, la Despersonalización es mayor en enfermeros; en España, es mayor en auxiliares/técnicos de enfermería. Se encontraron resultados estadísticamente significativos en la asociación de las dimensiones del *burnout* con las características sociodemográficas y laborales: edad, categoría profesional, lugar de trabajo, régimen laboral, turno de trabajo, duración de la experiencia laboral, tiempo trabajando en el mismo lugar y considerar el trabajo estresante.

**Conclusión:**

aunque los trabajadores de enfermería brasileños y españoles obtienen bajos niveles de Despersonalización y altos de Logro Profesional, existen niveles promedio de agotamiento emocional, lo que indica un importante factor preventivo en el que trabajar, ya que el agotamiento emocional se considera la primera etapa del *burnout*.

## Introduction

Mental disorders in nursing workers have become more expressive in the last decade, highlighting a serious problem in the field of occupational health and for health services in the international context^(^ 1 ^-^ 3 ^)^.

Among mental disorders, burnout, a psychosocial syndrome that arises in response to chronic work stressors, consisting of Emotional Exhaustion (EE), Depersonalization (DE) and Professional Achievement (PA)^(^ 4 ^)^, has been the subject of many investigations. Recent systematic review found that countries around the world are recognizing the impact of worker burnout and mental strain on productivity, especially the number of days lost and the impact on work ability^(^ 1 ^)^. Other studies indicate psychic disorders and burnout as responsible for most of the restrictions in nursing, intention to leave the profession, poor quality of care, increased errors, reflecting on patient safety^(^ 3 ^,^ 5 ^-^ 6 ^)^. 

Despite some peculiarities, the nursing work process happens similarly in different countries due to the inherent characteristics of care and its practical implications related to constant emotional tension, need for concentration, attention and great responsibility^(^ 7 ^)^. In addition, the nature of health work, characterized by the experience of pain, suffering and loss of patients, may affect workers, leading to the emergence of burnout syndrome^(^ 2 ^)^. 

However, burnout and other psychic disorders have been the target of investigations from the perspective of working conditions analysis and detailing of the variables that permeate this construct^(^ 8 ^-^ 11 ^)^. In this sense, organizational issues in Brazilian and European Nursing work have also been reflected in the physical and mental illness of workers due to adverse working conditions aggravated by the recent economic crisis, which exposed the workers of these scenarios to increased work overload, poor dimensioning of human resources, increased informal ties and fear of unemployment^(^ 8 ^,^ 10 ^,^ 12 ^)^.

These results have implications for the field of studies and practices in occupational health, revealing the need for protective measures to the mental health of health team professionals, especially Nursing.

In both Brazil and Spain, the Nursing team is made up of nurses, professionals with higher education, and nursing assistants / technicians, who professionalize with a shorter course, of medium level. The role of nurses involves more complex actions of care and team management, while assistants / technicians are responsible for the development of less complex activities and greater physical demands, such as patient hygiene and bed cleaning. By considering the data presented, the similarity of work contexts and the importance of diagnosing to intervene and prevent injuries to workers and, consequently, organizations and patients, this study aimed to analyze the scores of burnout dimensions in Brazilian and Spanish Nursing workers. 

## Method

Quantitative, cross-sectional and comparative study using non-probability convenience sampling consisting of 589 Brazilian and Spanish Nursing workers (nurses, nursing assistants/technicians). The ethical precepts in force in each country followed, and the study was approved in Brazil by the Research Ethics Committee of the University of São Paulo School of Nursing (opinion 912.483 of 11/17/2014) and in Spain by the Regional Ethics Committee Asturias Clinical Research Code 83/15). This research is part of the multicenter project entitled “From work contexts to occupational health of nursing professionals, a comparative study between Portugal, Brazil and Spain”, developed between the University of São Paulo School of Nursing, the University of Oviedo, the University of Porto and the Porto School of Nursing.

Data collection was performed by two of the authors, between June 2015 and December 2016, in four public hospitals and one pre-hospital in the north coast of São Paulo, Brazil, and in six hospitals and six pre-hospital institutions of a province of northern Spain. 

In both Brazil and Spain, the following data collection procedures were adopted: previously, the managers of the health institutions were verified the most favorable days and times for the invitation to Nursing workers to participate in the study; these were addressed individually in the workplace and, after clarification of the research content and relevant ethical aspects, the questionnaires, together with the Free and Informed Consent Term (FICT), They were distributed and subsequently collected, at a scheduled date and time, in a sealed envelope, without external identification, seeking to ensure the confidentiality and reliability of the information, as well as not interfering with the work routine. Nursing workers from the institutions that authorized the study application were eligible and were present at the workplace on the dates agreed for data collection with their managers; eight questionnaires with incomplete and / or illegible data was excluded.

For data collection, a Sociodemographic and Professional Characterization Questionnaire was used (which includes information on age, gender, marital status, professional category, length of professional training and current workplace, work regime and shift, considering the stressful work and has dependents to whom he cares) and the Maslach Burnout Inventory - Human Services Survey (MBI-HSS), prepared by Maslach and Jackson^(^ 13 ^)^, translated and validated into Portuguese^(^ 14 ^)^ and Spanish^(^ 15 ^)^. In both Brazil and Spain, the MBI-HSS consists of 22 items. In the Brazilian version, each item is distributed on a five-point scale, ranging from zero to four (“never” to “daily”), and in the Spanish version, on a seven-point scale, ranging from zero to six (“ never ”until“ daily ”). Thus, to enable the comparison of scores between countries, it was necessary to normalize them, which was performed by multiplying the scores of the Brazilian version by 600 and the Spanish version by 400. Then, the scores of both versions of the scale went from zero to 100. 

The MBI-HSS evaluates the worker’s experience in his work in three dimensions: Emotional Exhaustion (EE) (items 1, 2, 3, 6, 8, 13, 14, 16 and 20); Depersonalization (DE) (items 5, 10, 11, 15 and 22) and Professional Achievement (PA) (items 4, 7, 9, 12, 17, 18, 19 and 21). High values in the EE and DE dimensions, associated with low scores in the PA dimension, indicate burnout^(^ 14 ^)^. In this study, no cutoff was adopted: the analyzes were performed by calculating the mean scores in each dimension for both Brazilian and Spanish Nursing workers.

The collected data was entered into a spreadsheet of the Microsoft Office Excel® computer program, in the form of an electronic database, and later converted to the Statistical Package for the Social Sciences® (SPSS) 22.0 and free software R 3.3.2 for analysis. Descriptive and analytical analyzes of the data were performed using relative frequencies, absolute, mean, standard deviation, minimum and maximum, as well as association and correlation tests between the variables. To compare the mean burnout dimensions between countries, the Student’s t-test was used. In the ANOVA model, two factors were used to associate the burnout dimensions in each country with the categorical variables and the factorial ANCOVA for the association with the numerical variables, adopting a 95% confidence interval.

## Results

Of the 589 study participants, 47.20% are Brazilian and 52.80% are Spanish; 89.47% are female, and 60.61% live in stable marital union (Table 1). The average age is 39.5 years (SD 9.36; minimum 20; maximum 64). 

**Table 1:** Sociodemographic and professional characterization of nursing workers. Sao Paulo, Brazil, Asturias, Spain, 2015-2016

Variables		n*=589	%†
Country	Brazil	278	47,20
	Spain	311	52,80
			
Sex	Female	527	89,47
	Male	62	10,53
			
Marital Status	Single	177	30,05
	Stable Union	357	60,61
	Divorced, separated or widow	51	8,66
	Without information	4	0,68
			
Professional category	Nursing Auxiliary/Technician	286	48,56
	Nurse	303	51,44
			
Workplace	Pre-hospital care (SAMU‡)	34	5,77
	Hospital care	554	94,06
	Without information	1	0,17
			
Work Regime	With stability	148	25,13
	Without stability	436	74,02
	Without information	5	0,85
			
Work shift	Fixo	284	48,22
	Rotativo	279	47,37
	Sem informação	26	4,41
			
Do you have people depending on your care?	Fixed	183	31,07
	Rotary	342	58,06
	Without information	64	10,87
			
Do you consider your job stressful?	Yes	458	77,76
	No	122	20,71
	Without information	9	1,53

* n = number (absolute frequency);

† % = Percentage (relative frequency);

‡ SAMU = Emergency mobile care service

Regarding professional characteristics, 48.56% are mid-level nursing workers (technical) and 51.44% are nurses; 94.06% work in hospital care, 74.02% do not have job stability, and 77.76% consider work as stressful (Table 1). 

The average professional experience time is 13.5 years (SD 8.94; minimum 0.16; maximum 45) and average working time at the current workplace is 7.9 years (SD 7.05; minimum 0; maximum 40).

Regarding the dimensions of burnout, Spanish nursing workers had higher means in the DE dimension (p = 0.004) and Brazilian nursing workers had higher scores in the PA dimension (p = 0.031) (Table 2).

**Table 2:** Distribution and comparison of mean burnout dimensions in Brazilian and Spanish nursing workers. Sao Paulo, Brazil, Asturias, Spain, 2015-2016

Dimensions	Country	n*	M†	SD‡	Value - p§
Emotional Exaustion	Brazil	278	40	21	0,414
	Spain	308	42	19	
Depersonalization	Brazil	278	21	19	0,004
	Spain	310	26	19	
Professional Achievement	Brazil	278	74	18	0,031
	Spain	306	71	17	

* n = number (absolute frequency);

† M = Average;

‡ SD = Standard deviation;

§ Value-p (Student t tests)

There was no evidence of association of the burnout dimensions in each country with the categorical variables gender, marital status and presence of dependents. Table 3 presents the associations with statistical significance of the burnout dimensions with the other categorical variables. 

**Table 3:** Association of burnout dimensions in Brazilian and Spanish nursing workers with categorical variables. Sao Paulo, Brazil, Asturias, Spain, 2015-2016

Variables	Brazilians		Spanish		Total		Value - p*		
	n†	M‡(SD§)	n†	M‡(SD§)	n†	M‡(SD§)	Interation		ME||
Emotional Exhaustion									
Professional category									
NA/NT¶	168	40 (22)	75	47(18)	243	43(21)	0,112		0,029
Nurse	65	39 (18)	229	40(19)	294	40(19)			
Total	233	40 (21)	304	42(19)	537	41(20)			
Workplace									
PHC**	10	25(11)	22	21(20)	32	22(18)	0,382		<0,001
HC††	222	41(21)	282	43(18)	504	42(20)			
Total	232	40(21)	304	42(19)	536	41(20)			
Work shift									
Fixed	204	40(21)	41	30(26)	245	39(22)	0,001		0,807
Rotary	11	29(10)	260	43(18)	271	43(17)			
Total	215	40(21)	301	42(19)	516	41(20)			
Considers work stressful									
Yes	180	45(20)	246	45(18)	426	45(19)	0,939	>0,001	
No	49	24(16)	55	25(15)	104	25(15)			
Total	229	40(21)	301	42(19)	530	41(20)			
**Depersonalization**									
Professional category									
NA/NT¶	168	21(19)	75	29(19)	243	23(19)	0,024	0,960	
Nurse	65	25(18)	229	25(19)	294	25(19)			
Total	233	22(19)	304	26(19)	537	24(19)			
Workplace									
PHC**	10	12(11)	22	10(12)	32	10(12)	0,398	>0,001	
HC††	222	23(19)	282	27(19)	504	25(19)			
Total	232	22(19)	304	26(19)	536	24(19)			
Work shift									
Fixed	204	22(18)	41	17(21)	245	21(19)	0,047	0,380	
Rotary	11	18(14)	260	27(18)	271	27(18)			
Total	215	22(18)	301	26(19)	516	24(19)			
Considers work stressful									
Yes	180	25(19)	246	28(19)	426	27(19)	0,977	<0,001	
No	49	14(14)	55	17(15)	104	16(14)			
Total	229	22(19)	301	26(19)	530	24(19)			
**Professional Achievement**									
Work Regime									
With stability	17	63(19)	126	73(19)	143	72(19)	0,309	0,025	
Without stability	214	71(18)	176	76(16)	390	73(17)			
Total	231	71(18)	302	74(17)	533	73(17)			
Considers work stressful									
Yes	180	70(17)	246	73(18)	426	72(17)	0,272	0,041	
No	49	72(21)	55	79(14)	104	76(18)			
Total	229	71(18)	301	74(17)	530	73(17)			

* P-value (two-way ANOVA model);

† n = number (absolute frequency);

‡ M = Average;

§ SD = Standard deviation;

|| ME = Main effect;

¶ NA/ NT = Assistant/Nursing Technician;

** PHC = Prehospital Care;

†† HC = Hospital Care

In both Spain and Brazil, nursing assistants / technicians have higher EE than nurses (main effect p = 0.029); hospital care workers have higher levels of EE than prehospital care (main effect p <0.001) and participants who consider stressful work have higher levels of EE than those who do not consider stressful work (effect main p <0.001). In Brazil, workers with a fixed work shift have a higher degree of EE, while in Spain they are rotating shift workers (interaction p = 0.001).

The association of the DE dimension shows that in both Spain and Brazil, hospital care workers have higher levels than prehospital care (main effect p <0.001), just as workers who consider stressful work have higher ED levels than those not considering (main effect p <0.001). In Brazil, ED is higher in nurses, while in Spain it is higher in nursing assistants / technicians (interaction p = 0.024). In Brazil, workers with a fixed work shift have a higher degree of ED, while in Spain they are rotating shift workers (interaction p = 0.047) (Table 3). 

The data in Table 3 also shows that regarding the PR dimension, the association with job stability shows that in Spain and Brazil, workers without stability have higher PR (main effect p = 0.025), as well as workers who do not consider work stressful (main effect p = 0.041).

**Figure 1:**
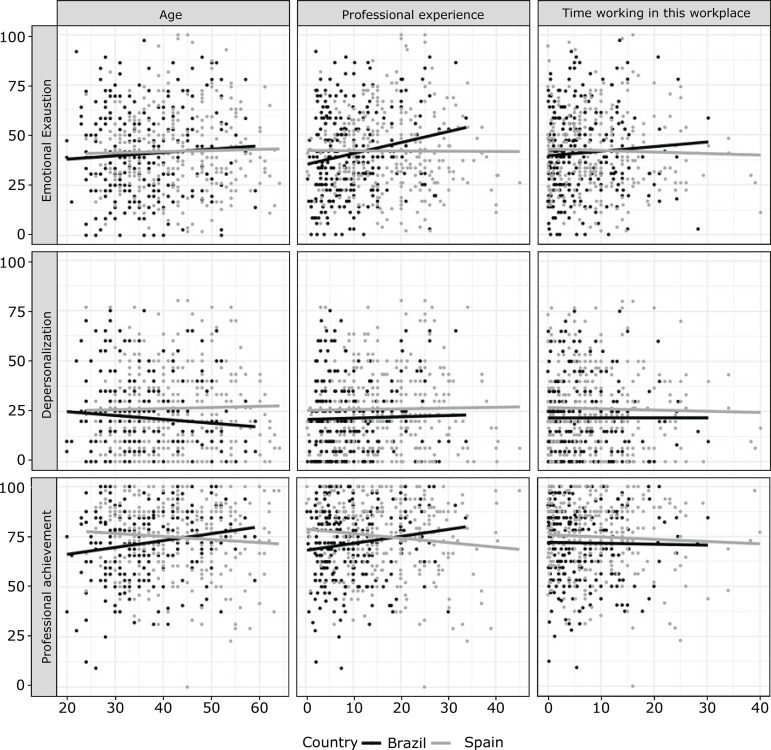
Relationship of the dimensions of burnout with the variables age, professional experience and length of work in the current workplace. Sao Paulo, Brazil, Asturias, Spain, 2015-2016

Figure 1 shows the association of burnout dimensions with numerical variables. No relationship was found between age and EE dimension (Regression Coefficient (CR) = 0.261; p-value interaction = 0.209; 95% Confidence Interval (CI): -0.14 to 0.66; Coefficient of Determination (R²) = 0.009). In Brazil, there is a positive correlation between the length of professional experience and EE, ie, as the length of professional experience increases, so does the level of EE (CR = 0.652; p-value interaction = 0.005; 95% CI: 0.19). a 1.10; R² = 0.023), whereas in Spain this interaction is not significant. 

In addition, the data indicate that in Brazil the longer the working time in the workplace, the higher the EE, while in Spain, the longer the working time in the workplace, the lower the EE (CR = 0.552; p-value interaction = 0.050; 95% CI: -0.001 to 1.106; R² = 0.010).

No relationship was found between the numerical variables age (CR = -0.086; p-value interaction = 0.661; 95% CI: -0.47 to 0.29; R² = 0.010), work experience (CR = 0.148; p-value interaction = 0.503; 95% CI: -0.28 to 0.58; R² = 0.012) and working time in the workplace (CR = 0.237; p-value interaction = 0.372; 95% CI: -0.28 to 0.75) ; R² = 0.010) with the dimension DE.

When associating the PA dimension with the numerical variables, a positive correlation was observed between age and PA in the Brazilian workers, and a negative correlation in the Spanish workers (CR = 0.475; p-value interaction = 0.008; 95% CI: 0.12 to 0.82). (R 2 = 0.023); In Brazil there is a positive correlation between professional experience and PA, while in Spain this correlation is negative (CR = 0.515; p-interaction value = 0.012; 95% CI: 0.11 to 0.91; R² = 0.024). There was no correlation between working time in the workplace and PA (CR = 0.025; p-interaction value = 0.920; 95% CI: - 0.46 to 0.51; R² = 0.012).

## Discussion

Currently, few studies analyze burnout syndrome in nursing workers from a multicultural perspective. The sample of this study consisted of Brazilian and Spanish young adult workers, with female predominance, corroborating the results obtained in other studies ^(^ 16 ^-^ 19 ^)^. This study also demonstrates that the technical division of labor, the lack of job stability and the perception that work is stressful are factors that permeate the Brazilian and Spanish scenario. 

There was a great variation in the international literature when comparing, in Nursing workers, the averages of each dimension of burnout, even within the countries studied here^(^ 2 ^,^ 17 ^,^ 20 ^-^ 21 ^)^. In this study, when analyzing the mean burnout dimensions in Brazilian and Spanish nursing workers, it is observed that, despite low DE and high PR levels, there are average EE levels, indicating an important preventive factor to be worked on, since EE is considered the first stage of burnout, leading to ED and may interfere with PR. The literature shows that EE is a result of work overload and conflicts in interpersonal relationships and is related to the lack of energy to perform work activities, the feeling of being overloaded, fatigued and with physical and mental exhaustion. ED is a form of coping with EE, an attempt to distance oneself from the recipients of one’s work due to EE, which leads the worker to a negative response in the interpersonal context, treating the recipients of his work with cynicism or coldness as if were objects, such as those responsible for their problems, and the reduced PA results from the self-assessment process, when the worker feels incompetent, failed, low self-esteem and poor work performance^(^ 4 ^)^. 

In addition, EE is associated with job dissatisfaction, intention to quit next year and feeling overworked^(^ 17 ^)^.

Increasingly, when it comes to the organization of work, the influence of the leader in maintaining a work and team climate conducive to the achievement of workers and the achievement of organizational goals is becoming increasingly evident. In this context, the continuous formation of leaders and the team’s maturity may represent a key point for the construction of strategies to promote quality of life at work and, consequently, reduction of burnout.

The comparison of the burnout dimensions between countries showed significant differences, where Brazilian nursing workers had higher PA, while Spanish nursing workers had higher DE. 

Another observed difference has to do with work organization so that EE and DE are higher in Brazilian Nursing workers with a fixed work shift, whereas in Spain this occurs in the rotating shift.

The professional category also seems to play an important role in the different dimensions of burnout by country. This study observed that EE levels were higher in Nursing assistants / technicians than in nurses, both in Brazil and Spain. It is important to highlight the technical and social division of Nursing work, which not only exposes workers to different types of workloads but also to different sickening processes, since higher-level workers are dedicated to the activities of greater intellectual and social demands management and mid-level workers to manual activities that require more physical effort. 

In the Brazilian and Spanish contexts, the duties of Nursing assistants / technicians include direct patient care activities, such as cleaning and hygiene techniques, causing greater physical wear to the category. On the other hand, direct pressure during strict supervision and quality of care, both by users and by the supervisor (nurse), is also an element capable of increasing EE in these workers.

This data differs from research conducted with Italian hospital health workers ^(^ 22 ^)^, but corroborates a Brazilian study that showed high levels of EE associated with low educational level^(^ 23 ^)^. 

Regarding the higher levels of DE in Brazilian nurses than in Nursing assistants / technicians, the data coincide with the results of a survey (22) conducted in Italy and a study^(^ 23 ^)^ of Brazilian hospital health workers that found an association of DE. with higher educational level in nurses. It is assumed that the higher level of education is linked to the attribution of higher responsibilities and higher expectations of these workers in relation to the profession. However, another study was consistent with results in the Spanish population, which showed higher levels of DE in nursing technicians / assistants^(^ 24 ^)^. 

In both Spain and Brazil, the results indicated higher levels of EE and DE in hospital care nursing workers. It seems possible that these results are due to the peculiarities of the work environment: while the pre-hospital is characterized by greater dynamism and freedom in the decisions themselves, in the hospital, the work is done in closed units, with patients hospitalized for long periods of time in continuous and direct contact with supervisors and managers, and more frequently performing administrative tasks.

Considering stressful work showed an association with burnout, since these nursing workers had higher levels of EE and DE, and lower PR, corroborating the literature, which indicates stress as a predictor of burnout^(^ 22 ^,^ 25 ^)^. Other authors found an association between stress and two of the burnout dimensions: EE and DE^(^ 26 ^-^ 28 ^)^.

The data also showed that Brazilian and Spanish workers without stability have higher levels of PR. While on the one hand, instability may be linked to the uncertainty of not having a stable job, on the other, job stability may imply lower expectations for professional growth. The association and comparison of EE with job stability in this study were not significant, contrasting a study with hospital health workers in Brazil, where higher EE scores were found in workers with stability than in those without stability^(^ 29 ^)^.

Moreover, the results showed that, contrary to what happens in Brazil, Spain, younger nursing workers with shorter professional experience have lower PR. These results are similar to those previously found in the occupational environment^(^ 22 ^,^ 30 ^)^. Thus, another Brazilian study with hospital health workers also found an association between older age and higher PR^(^ 29 ^)^.

In Brazil, as the length of professional experience increases, so do the levels of EE, while in Spain this correlation is nil. The influence of working time in the same workplace also points to contradictory data: while in Spain, working in the same workplace for a longer time is associated with lower EE, in Brazil, the opposite occurs, collaborating with the study conducted in Italy^(^ 22 ^)^. This may be due to Spanish Nursing workers developing a more effective work adaptation process and not accumulating time responsibilities in the same workplace.

Considering the data related to both contexts, the need to implement intervention measures to reduce the risks of burnout development is evident. In this regard, the studies reinforce greater effectiveness and durability of interventions that address both the individual and organizational levels. The individual approach includes psychoeducational actions, with discussion of risk factors, relaxation practices, development of coping strategies, among others ^(^ 31 ^-^ 32 ^)^. At the organizational level, interventions interfere with working conditions, as can be seen in the study with Australian nurses, which evaluated the impact of an organizational intervention in reducing occupational stress, using a tool to assess workloads, increasing staff numbers, access to professional development, among others, and achieved a significant reduction in psychological distress and emotional exhaustion and a significant improvement in job satisfaction^(^ 33 ^)^.

It is worth highlighting teamwork as a health strengthening for nursing workers, given that it can provide a collaborative practice in which roles are well defined and there is a focus to be achieved, although there is the specificity of each action. From this perspective, it is essential to involve the subjects in the process of construction and redesign of the work with a view to improving the work climate, team climate and, consequently, valuing professionals.

The limitations of this study are related to its design, which makes the cause and effect relationship impossible, the number of participants and the lack of some variables that may interfere with burnout levels, such as resilience^(^ 34 ^-^ 35 ^)^, which can lead the subject to act positively in the face of adversity, becoming a protective factor in the development of this problem. It may be interesting that the study also includes other health workers, including those in primary care, considering the various scenarios of action and their relevance to health at the international level. However, the data show similarities in the contexts and support the planning of actions at the individual and collective levels in the face of illness at work and the need to maintain patient quality and safety.

## Conclusion

In conclusion, despite cultural, economic and social differences, Nursing workers face similar problems, with some particularities. Brazilian and Spanish nursing workers have moderate levels of EE, low DE levels and high PA. When comparing the populations, it was observed that the Brazilians had higher PA averages and the Spanish higher DE averages. It was also verified that dimensions of burnout are associated with some sociodemographic and work characteristics, such as: age; professional category; workplace; work regime; work shift; time of professional experience; working time in the same workplace and consider stressful work.

The investigation of burnout in nursing workers and its associated factors, as well as prevention and coping mechanisms, becomes indispensable as an instrument of evaluation and support for the implementation of preventive and interventional measures, seeking to protect the health of the worker and, consequently, patient safety and organizational success.
